# Change in level of productivity in the treatment of schizophrenia with olanzapine or other antipsychotics

**DOI:** 10.1186/1471-244X-11-87

**Published:** 2011-05-17

**Authors:** Hong Liu-Seifert, Haya Ascher-Svanum, Olawale Osuntokun, Kai Yu Jen, Juan Carlos Gomez

**Affiliations:** 1 Lilly Research Laboratories, Indianapolis, Indiana, USA

## Abstract

**Background:**

When treating schizophrenia, improving patients' productivity level is a major goal considering schizophrenia is a leading cause of functional disability. Productivity level has been identified as the most preferred treatment outcome by patients with schizophrenia. However, little has been done to systematically investigate productivity levels in schizophrenia. We set out to better understand the change in productivity level among chronically ill patients with schizophrenia treated with olanzapine compared with other antipsychotic medications. We also assessed the links between productivity level and other clinical outcomes.

**Methods:**

This post hoc analysis used data from 6 randomized, double-blind clinical trials of patients with schizophrenia or schizoaffective disorder, with each trial being of approximately 6 months duration. Change in productivity level was compared between olanzapine-treated patients (HGBG, n = 172; HGHJ, n = 277; HGJB, n = 171; HGLB, n = 281; HGGN, n = 159; HGDH, n = 131) and patients treated with other antipsychotic medications (separately vs. haloperidol [HGGN, n = 97; HGDH, n = 132], risperidone [HGBG, n = 167; HGGN, n = 158], quetiapine [HGJB, n = 175], ziprasidone [HGHJ, n = 271] and aripiprazole [HGLB, n = 285]). Productivity was defined as functional activities/work including working for pay, studying, housekeeping and volunteer work. Productivity level in the prior 3 months was assessed on a 5-point scale ranging from no useful functioning to functional activity/work 75% to 100% of the time.

**Results:**

Chronically ill patients treated with olanzapine (OLZ) experienced significantly greater improvement in productivity when compared to patients treated with risperidone (RISP) (OLZ = 0.22 ± 1.19, RISP = -0.03 ± 1.17, p = 0.033) or ziprasidone (ZIP) (OLZ = 0.50 ± 1.38, ZIP = 0.25 ± 1.27, p = 0.026), but did not significantly differ from the quetiapine, aripiprazole or haloperidol treatment groups. Among first episode patients, OLZ therapy was associated with greater improvements in productivity levels compared to haloperidol (HAL), during the acute phase (OLZ = -0.31 ± 1.59, HAL = -0.69 ± 1.56, p = 0.011) and over the long-term (OLZ = 0.10 ± 1.50, HAL = -0.32 ± 1.91, p = 0.008). Significantly more chronically ill and first episode patients treated with olanzapine showed moderately high (>50%-75% of the time) and high levels of productivity (>75%-100% of the time) at endpoint, when compared to risperidone or haloperidol-treated patients (p < .05), respectively. Higher productivity level was associated with significantly higher study completion rates and better scores on the positive, negative, disorganized thoughts, hostility and depression subscales of the Positive and Negative Symptom Scale (PANSS).

**Conclusions:**

Some antipsychotic medications significantly differed in beneficial impact on productivity level in the long-term treatment of patients with schizophrenia. Findings further highlight the link between clinical and functional outcomes, showing significant associations between higher productivity, lower symptom severity and better persistence on therapy.

**Trial Registration:**

clinicaltrials.gov identifier NCT00088049; NCT00036088

## Background

Schizophrenia is a severe and lifelong mental illness characterized by impairment of most domains of cognitive functioning, often leading to functional disability [1]. Patients with schizophrenia suffer not only from symptoms such as delusions or hallucinations but also impaired occupational functioning and low levels of productivity (e.g., paid employment, being a student, or other useful activity) and high rates of unemployment [2-4].

The poor productivity level among patients with schizophrenia has long been recognized as a core component of the burden of illness and its economic cost [5,6]. The financial cost of schizophrenia in the United States in 2002 was estimated to be $62.7 billion [6]. In another study of the economic burden of schizophrenia in the United States in 2002, the indirect excess cost due to unemployment was found to be the largest component of the overall excess annual costs [7].

Improving patients' productivity level is an important goal in the treatment of schizophrenia and was previously identified as the most preferred treatment outcome, more than improvement of symptoms, by clinicians, patients, their families as well as public policy makers [8]. Rosenheck et al. (2005) [9] evaluated the personal outcome preferences of a large sample of patients treated for schizophrenia and identified work as the 4th preferred outcome among 6 assessed domains including social life, energy, symptoms, work, confusion and treatment-emergent adverse events. Importantly, several clinical guidelines [10-12] cite supported employment programs as one of the most valuable psychosocial treatment interventions for schizophrenia.

Although little is known about predictors of productivity level in the treatment of patients with schizophrenia, the link between medication adherence or persistence and functional outcomes has been consistently shown [13-15]. Adherence to antipsychotic treatment is associated with better long-term improvements in outcome measures including decreased risk of psychiatric hospitalizations, detentions, victimizations, substance use, and severity of alcohol-related issues, as well as improvements in mental health and satisfaction with social life in general [14]. In addition, longer treatment duration with antipsychotics (persistence) was found to be associated with improved symptom severity levels [16] and greater functional outcomes in the treatment of patients with schizophrenia [15]. Recent meta-analyses have shown that antipsychotics significantly differ on their pharmacology, efficacy, safety and tolerability profiles [17,18]. The choice of antipsychotics may also play a significant role in patients' adherence to or persistence with antipsychotic medications, as adherence and persistence on antipsychotic medication appear to be highly intercorrelated [19]. Olanzapine treatment is associated with better persistence, or lower rates of medication discontinuation for any cause, compared to other antipsychotics [20-24]. Moreover, few studies have suggested that this advantage may be due to the greater efficacy of olanzapine relative to other antipsychotics [21,22]. However, it is unclear whether these differences have any impact on the patient's productivity level.

Taken together, productivity is a very important area in the treatment of schizophrenia and yet it is largely unstudied. To our knowledge, there has not been any systematic investigation on the comparative productivity among antipsychotic drugs. To address this question, we conducted a post hoc analysis of double-blind, active-controlled trials from Lilly clinical trial database comparing olanzapine with other antipsychotic drugs on change in level of productivity. The links between productivity and symptom severity and between productivity and patients' persistence on therapy were also investigated.

## Methods

### Data source

A post hoc analysis of six randomized, double-blind clinical trials of patients with schizophrenia or schizoaffective disorders was performed. Participants from 5 randomized clinical trials were chronically ill, whereas participants in one study were patients experiencing their first schizophrenic episode (i.e., "first episode patients"). Trials that studied chronically ill patients ranged between 22 and 28 weeks in duration. The study of first episode patients included an acute phase (first 12 weeks) and the longer-term phase (the following 24 weeks). These 6 studies have been previously published comparing olanzapine with risperidone (HGBG, OLZ: 10 to 20 mg/day; RISP: 4 to 12 mg/day) [25], quetiapine (HGJB, OLZ: 10 to 20 mg/day; QUE: 300 to 700 mg/day) [26], ziprasidone (HGHJ, OLZ: 10 to 20 mg/day; ZIP: 80 to 160 mg/day) [27], Aripiprazole (HGLB, OLZ: 15 to 20 mg/day; ARI: 15 to 30 mg/day) [28] and haloperidol (HGDH: acute treatment phase, the initial dose titration ranges for the first 6 weeks were OLZ [5 to 10 mg/day] and HAL [l2 to 6 mg/day; for the second 6 weeks of the acute phase and for the entire continuation phase, the allowed doses were OLZ [5 to 20 mg/day and haloperidol 2 to 20 mg/day]) [29-31]. Table 1 presents these studies, their sample sizes and the study duration.

**Table 1:** Summary of Baseline Demographics and PANSS Total Scores in Patients with Schizophrenia

Study	*HGBG*		*HGHJ*		*HGJB*		*HGLB*		*HGGN*			*HGDN*	
	**OLZ**	**RISP**	**OLZ**	**ZIP**	**OLZ**	**QUET**	**OLZ**	**ARI**	**OLZ**	**RISP**	**HAL**	**OLZ**	**HAL**
**N**	**172**	**167**	**277**	**271**	**171**	**175**	**281**	**285**	**159**	**158**	**97**	**131**	**132**

***Characteristics***													
Age, Mean	36.02	36.41	40.05	38.24	41.67	40.45	38.3	37.3	38.40	39.5	39.8	23.53	24.00
(SD), y	(10.81)	(10.6)	(11.59)	(12.1)	(9.53)	(9.6)	(10.50)	(10.4)	(7.90)	(8.25)	(8.32)	(4.61)	(4.90)

***Gender (%)***													
Male	114 (66.3)	106 (63.5)	180 (65.0)	172 (63.5)	114 (66.7)	113 (65.1)	194 (69.0)	190 (66.7)	115 (72.3)	111 (70.3)	69 (71.1)	104 (79.4)	111 (84.1)
Female	58 (33.7)	61 (36.5)	97 (35.0)	99 (36.5)	57 (33.3)	58 (34.9)	87 (31.0)	95 (33.3)	44 (27.7)	47 (29.7)	28 (28.9)	27 (20.6)	21 (15.9)

***Race (%)***													
Caucasian	129 (75.0)	124 (74.3)	115 (41.5)	124 (45.8)	90 (53.2)	88 (50.3)	78 (27.8)	90 (31.6)	95 (59.7)	101 (63.9)	51 (52.6)	67 (51.1)	72 (54.5)
African	35 (20.3)	36 (21.6)	78 (28.2)	66 (24.4)	64 (37.4)	65 (37.1)	87 (31.0)	90 (31.6)	43 (27.0)	43 (27.2)	31 (32.0)	49 (37.4)	50 (37.9)
Asian	1 (0.6)	1 (0.6)	2 (0.7)	3 (1.1)	0	0	0	0	5 (3.2)	2 (1.3)	1 (1.0)	4 (3.1)	5 (3.8)
Hispanic	3 (1.7)	8 (6.1)	1 (0.4)	0	13 (7.6)	17 (9.7)	96 (34.2)	84 (29.5)	13 (8.2)	6 (3.8)	9 (9.3)	8 (6.1)	4 (3.0)
Other	4 (2.3)	4 (2.4)	63 (22.7)	61 (22.5)	3 (1.8)	5 (2.9)	20 (7.1)	19 (6.7)	3 (1.9)	6 (3.8)	5 (5.2)	3 (2.3)	1 (0.8)

PANSS Total Score Mean (SD)	96.3 (17.0)	95.7 (16.2)	99.5 (18.4)	101.8 (21.1)	84.1 (14.8)	85.2 (4.7)	95.7 (15.9)	95.0 (15.4)	82.6 (13.1)	84.1 (14.7)	82.7 (14.1)	81.0 (14.5)	82.5 (17.5)

*NOTE: HGJB was 22 weeks; HGGN was 24 weeks; HGBG, HGHJ and HGLB were 28 weeks*.

### Outcome measures

Change from baseline to endpoint in productivity level was compared between olanzapine and other antipsychotic treatment groups (separately versus haloperidol, risperidone, quetiapine, ziprasidone and aripiprazole). Productivity was defined as functional activities/work (useful work) including working for pay, being a student, housekeeping, and volunteer work in the past 3 months. Productivity level was assessed by study investigators on a 5-point scale: 1. No useful functioning, 2. > 0 to 25% of the time, 3. > 25% to 50% of the time, 4. > 50% to 75% of the time, 5. > 75% to 100% of the time.

Symptom severity was measured by 5 Positive and Negative Symptom Scale (PANSS) factor scales: positive, negative, disorganized thoughts, hostility and depression [32].

### Statistical Analysis

Change from baseline to endpoint in productivity level was compared between olanzapine and each of the other antipsychotic medications within the individual study based on an Analysis of Covariance (ANCOVA) model with terms for baseline productivity score and treatment. Percentage of patients with > 50% to ≤75% ("moderately high") or > 75% ("high") productivity level at endpoint was also compared between treatment groups within each study using Fisher's exact test.

The association between productivity level and treatment persistence was assessed using an ANCOVA model which included terms for baseline productivity score and early treatment discontinuation status (Y/N). In addition, this post hoc analysis used Pearson correlations to assess the relationship between clinical outcomes, measured by the 5 PANSS factors--positive, negative, disorganized thoughts, hostility and depression; and the productivity level at the end of the study.

All statistical tests were based on a 2-tailed significance level of 0.05.

## Results

### Patient Baseline Characteristics

Table 1 shows baseline clinical and demographic characteristics for patients in each of the 6 studies used in the analysis. The majority of the patients were male and Caucasian. Mean baseline PANSS scores (range = 81.0-101.8) reflected moderate or greater illness severity for most of the patients.

### Productivity and chronically ill patients

#### Change in level of productivity

**Figure 1:**
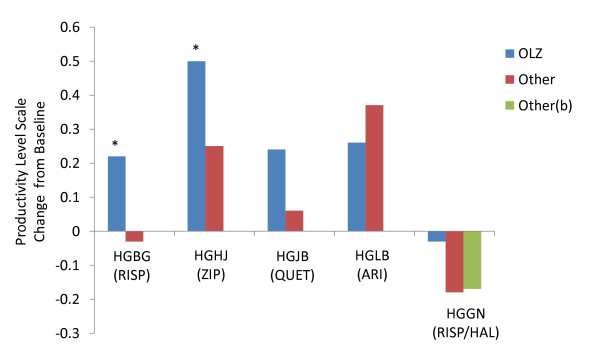
**By-study comparison of baseline-to-endpoint change in productivity level in olanzapine-treated versus other-treated chronically ill patients with schizophrenia**. Comparison between olanzapine and each of the other antipsychotics--aripiprazole, haloperidol, risperidone, quetiapine, and ziprasidone--demonstrated that olanzapine was consistently associated with higher mean changes in baseline-to-endpoint productivity level. Productivity level was assessed by study investigators on a 5-point scale, with scale scores corresponding to how often functional activities can be performed: 1, no useful functioning; 2, > 0% to ≤25% of the time; 3, > 25% to ≤50% of the time; 4, > 50% to ≤75% of the time; and 5, >75% to ≤100% of the time. Abbreviations: ARI = aripiprazole, HAL = haloperidol, OLZ = olanzapine, RIS = risperidone, QUE = quetiapine, ZIP = ziprasidone. *HGBG, HGHJ -p < .05.

Baseline-to-endpoint mean change in productivity level scores were significantly greater for olanzapine-treated patients compared to patients treated with risperidone in HGBG or ziprasidone (p < .05). Olanzapine-treated patients did not significantly differ from quetiapine-, aripiprazole- and haloperidol-treated patients (Figure 1).

**Figure 2:**
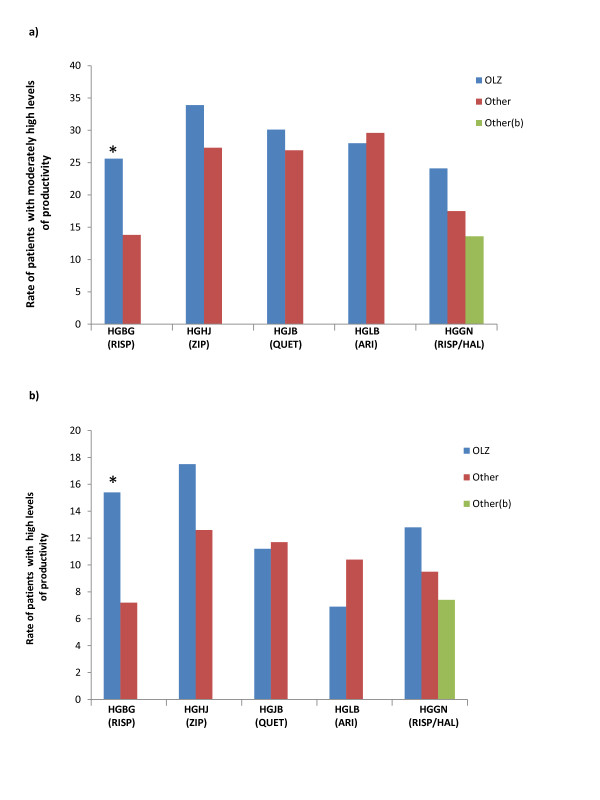
**By-study comparison of endpoint productivity level in olanzapine-treated versus other-treated chronically ill patients with schizophrenia**. Comparison between olanzapine and each of the other antipsychotics--aripiprazole, haloperidol, risperidone, quetiapine, and ziprasidone, more patients treated with olanzapine consistently had moderately high (>50% to 75% of the time) (a) and high productivity (>75% to 100% of the time) (b) at endpoint. Abbreviations: ARI = apripiprazole, HAL = haloperidol OLZ = olanzapine, RIS = risperidone, QUE = quetiapine, ZIP = ziprasidone. *HGBG -p < .05.

At endpoint, olanzapine-treated patients had significantly higher rates of moderately high and high levels of productivity (Figure 2) than risperidone-treated patients in HGBG (p < .05), but did not significantly differ on these measures from the ziprasidone, quetiapine, aripiprazole or haloperidol treatment groups.

#### Level of productivity and symptom severity

Pearson correlations between each of the 5 PANSS factor scale scores and productivity level at the end of the study are shown in Tables 2 and 3. Correlations between productivity level and PANSS positive, negative, disorganized thoughts, hostility and depression were significant for all studies (p < .05) except for HGJB, in which productivity level was not statistically significantly associated with symptoms of hostility (.099, p < .091) or depression (.039, p < .502). The magnitude of the correlation coefficients ranged from 0.039 (productivity level and depression; HGJB) to -0.471 (productivity level and disorganized thoughts; HGHJ).

**Table 2:** Pearson Correlations of Productivity Level with PANSS Factor Scores at Endpoint - Chronically Ill Patients

*PANSS Factors*	*HGBG* (n = 307)	*HGHJ* (n = 507)	*HGJB* (n = 288)	*HGLB* (n = 516)	*HGGN* (n = 358)
Negative Symptoms	-0.34 [p < .0001]	-0.447 [p < .0001]	-0.31 [p < .0001]	-0.262 [p < .0001]	-0.291 [p < .0001]
Positive Symptoms	-0.37 [p < .0001]	-0.423 [p < .0001]	-0.217 [p < .0002]	-0.306 [p < .0001]	-0.25 [p < .0001]
Disorganized Thoughts	-0.328 [p < .0001]	-0.471 [p < .0001]	-0.339 [p < .0001]	-0.334 [p < .0001]	-0.363 [p < .0001]
Hostility	-0.356 [p < .0001]	-0.292 [p < .0001]	-0.099 [p < 0.091]	-0.245 [p < .0001]	-0.159 [p < .0025]
Depression	-0.184 [p=.0012]	-0.202 [p < .0001]	0.039 [p < 0.502]	-0.185 [p < .0001]	-0.114 [p < .0308]

NOTE: HGBG, HGHJ, HGJB, HGLB, and HGGN = Study Codes

**Table 3:** Pearson Correlations of Productivity Level with PANSS Factor Scores at Endpoint - First Episode Patients

*PANSS Factors*	*HGDH-Acute* *(n = 221)*	*HGDH-Long-Term* *(n = 221)*
Negative Symptoms	-0.347 [p < .0001]	-0.502 [p < .0001]

Positive Symptoms	-0.414 [p < .0001]	-0.493 [p < .0001]

Disorganized Thoughts	-0.374 [p < .0001]	-0.474 [p < .0001]

Hostility	-0.22 [p < .0001]	-0.318 [p < .0001]

Depression	-0.177 [p < .0001]	-0.296 [p < .0001]

NOTE: HGDH = Study Code

#### Treatment persistence and productivity level

**Figure 3:**
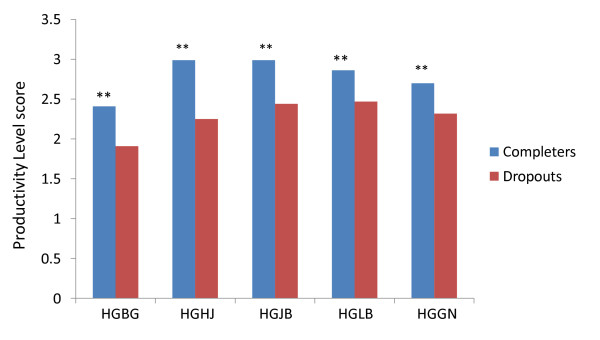
**By-study comparison of mean productivity score of completers versus dropouts in chronically ill patients with schizophrenia treated with antipsychotics**. Comparison between completers and dropouts showed that chronically ill patients who completed the studies had better productivity levels in each of the 5 studies. Abbreviations: ARI = aripiprazole, HAL = haloperidol, OLZ = olanzapine, RIS = risperidone, QUE = quetiapine, ZIP = ziprasidone. ** p < .001.

Productivity level scores for study completers versus dropouts (measuring treatment persistence) are shown in Figure 3. Chronically ill patients who completed the studies had statistically significantly better productivity levels compared to dropouts in each of the 6 studies (p < .001).

### Productivity and first episode patients

#### First episode patients change in level of productivity

**Figure 4:**
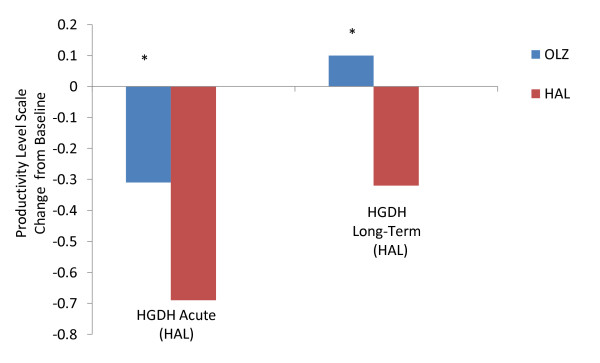
**Comparison of baseline-to-endpoint change in productivity level in olanzapine-treated versus haloperidol-treated first episode patients with schizophrenia**. Comparison between olanzapine and haloperidol demonstrated that olanzapine was consistently associated with higher mean changes in baseline-to-endpoint productivity level for both acute phase (first 12 weeks) and long-term phase (the following 24 weeks) treatment. Productivity level was assessed by study investigators on a 5-point scale, with scale scores corresponding to how often functional activities can be performed: 1, no useful functioning; 2, > 0% to ≤25% of the time; 3, > 25% to ≤50% of the time; 4, >50% to ≤75% of the time; and 5, >75% to ≤100% of the time. Abbreviations: HAL = haloperidol, OLZ = olanzapine. *HGDH-Acute, HGDH-Long-Term -p < .05.

Olanzapine-treated patients showed significantly greater baseline-to-endpoint change in productivity level compared to haloperidol treated patients (p < .05) (Figure 4). This was observed in the acute phase (12 weeks) and over the longer-term (24 weeks). At endpoint, olanzapine-treated patients showed significantly higher rates of moderately high and high levels of productivity (data not shown) than haloperidol-treated ones (p < .05).

#### Level of productivity and symptom severity

Pearson correlations between each of the 5 PANSS factor scale scores and productivity level at the end of the study (Tables 2 and 3) were statistically significant for both the acute phase and the longer-term treatment phases (p < .001) with correlation coefficients ranging between -0.177 (productivity level and depression in the acute phase) and -0.502 (productivity level and negative symptoms in the long-term phase).

#### Treatment persistence and productivity level

Study completers of the acute and long-term treatment phase had statistically significantly better productivity level scores compared to study dropouts (p < .05) (data not shown).

## Discussion

This is the first study to evaluate improvement in productivity among patients with schizophrenia treated with various antipsychotics. Using data from 6 randomized, double-blind clinical trials of antipsychotic therapy, this post hoc investigation detected significant differences between olanzapine and some of the studied antipsychotics on improvement in level of productivity. Change in level of productivity from baseline was significantly greater in both chronically ill and first episode patients with schizophrenia treated with olanzapine compared with some of the other antipsychotics. In chronically ill patients, olanzapine treatment was associated with significantly greater improvement in productivity levels compared to risperidone and ziprasidone, but not with quetiapine or aripiprazole. Higher rates of moderately high and high productivity levels at endpoint were also observed with olanzapine- compared to risperidone treatment. For first episode patients treated with olanzapine, significantly higher rates of moderately high and high productivity at endpoint were observed during the acute phase and the long-term treatment phase compared to haloperidol-treated patients.

Current findings are consistent with those reported in a randomized, open label, flexible dose, multi-center study of outpatients with schizophrenia who were assigned to a 1-year treatment with olanzapine or risperidone [33]. In that study, the greatest treatment group difference was found on the occupational/employment outcome measure (p = 0.0024). The present findings are, however, inconsistent with two other schizophrenia studies in which the olanzapine- and risperidone-treated patients did not significantly differ on employment outcomes [34] or job skills learning [35]. This inconsistency may be related to methodological differences and especially to differences in the definition of productivity. For example, we used an ordinal measure of productivity, which encompassed work for pay as well as useful non-paid activity such as volunteer work or being a student, thus possibly being more sensitive to change than the dichotomous measure (i.e., working for pay vs. not working) used previously [33].

We also found significant associations between productivity level and improvement in symptom severity levels among chronically ill and first episode patients. These findings are consistent with previous schizophrenia research in which symptom improvement and symptom remission were shown to be associated with better functional outcomes [36-39]. While our study found significant associations between productivity level and disorganized thinking, positive symptoms and negative symptoms, previous research has shown that negative symptoms have a more robust link to functional outcomes, with lower PANSS negative symptoms being able to predict functional remission [36] and paid employment [40-43]. Conversely, poorer employment and occupational functioning were previously found to be strongly predicted by severe negative symptoms [40,42,43].

Our analysis also found significant associations between productivity level and persistence on therapy, defined as study completion. In each of the 6 trials, which ranged between 12 (for acute phase) and 28 weeks in duration, the completers had significantly greater improvement in productivity level than patients who dropped out of the study. These results are consistent with previous studies showing that longer duration of antipsychotic treatment is correlated with better functional outcomes, including better occupational functioning, among patients with schizophrenia [15].

The current study found an advantage for olanzapine therapy on improving productively level compared to treatment with risperidone, ziprasidone, and haloperidol, but not when compared with quetiapine and aripiprazole. Although our post hoc exploratory analysis cannot clarify the underlying drivers of the current results, the findings can be explained using the link previously shown between longer treatment duration and better clinical efficacy in the treatment of patients with schizophrenia [21,22,44-46]. In meta-analytical studies of antipsychotic therapy for schizophrenia, which incorporated data from numerous randomized clinical trials, olanzapine was found to confer greater efficacy compared to haloperidol [17] risperidone and ziprasidone [18]. Furthermore, patients treated with olanzapine were consistently found to stay longer on treatment compared to those treated with haloperidol [23,31,44-49], risperidone [21,23,24,28,48-56] and ziprasidone [21,27,57-59]. Thus, antipsychotic treatment choice may influence patients' improvement in symptom severity, their treatment duration and their functional outcomes as measured, in this study, by productivity levels.

This study possesses several limitations. First, this is a post hoc analysis of 6 randomized, double-blind clinical trials composed of chronically ill patients with schizophrenia and first episode schizophrenic patients studied over different treatment durations [between 12 (for acute phase) and 28 weeks]. The current findings will require replication in studies assessing these outcomes in an a priori manner. Second, the current analysis was conducted in randomized clinical trials, thus it is unclear if the findings may generalize to schizophrenia patients treated in usual care settings. Lastly, as this research is the first to systematically investigate the productivity levels in treatment of schizophrenia, patients' productivity level was assessed with a single item with 5 response options and the reliability and validity of this productivity measure has not been established yet.

## Conclusions

Current findings suggest that antipsychotic medications may significantly differ on beneficial impact on productivity level in the treatment of patients with schizophrenia, and highlight the link between clinical and functional outcomes, showing significant associations between higher productivity, lower symptom severity and better persistence on therapy. This post hoc analysis suggests an advantage for olanzapine therapy over several other antipsychotics on improving productivity levels among chronically ill and first episode patients with schizophrenia. This finding will require replication in future research.

## Competing interests

Drs. Liu-Seifert, Ascher-Svanum, Osuntokun, Jen and Gomez are employees of Eli Lilly.

## Authors' contributions

HL-S, HA-S, OO, and J-CG contributed to the conception and design, as well as the acquisition of the data. Additionally, HL-S and HA-S contributed to the analysis of the data. KYJ drafted the manuscript. All authors contributed to the interpretation of the data, revised and edited the manuscript critically for important intellectual content, and gave final approval of the version to be published.

## Pre-publication history

The pre-publication history for this paper can be accessed here:

http://www.biomedcentral.com/1471-244X/11/87/prepub
